# Origin, Selection and Current Status of the Utrerana Chicken Breed: A Review

**DOI:** 10.3390/ani13182982

**Published:** 2023-09-21

**Authors:** Antonio Plata-Casado, Carmelo García-Romero, Pedro González-Redondo

**Affiliations:** 1 Consultancy in Organic Agriculture and Holistic Management, 41710 Utrera, Spain; asusl@hotmail.com; 2 Real Academia de Ciencias Veterinarias, Instituto de España, 28006 Madrid, Spain; guindalejocarmelo@gmail.com; 3 Departamento de Agronomía, Universidad de Sevilla, Carretera de Utrera Km 1, 41013 Seville, Spain

**Keywords:** animal genetic resources, native breed, conservation programs, laying hen, poultry

## Abstract

**Simple Summary:**

The Utrerana chicken is an endangered breed native to Southern Spain. Its constitution and selection by a poultry farmer began in Utrera (Seville province) in the mid-1920s starting from backyard hens that laid large eggs. By the 1950s, the breed achieved an average laying yield of 180 eggs per hen and year, an optimal performance that allowed it to compete with other breeds of laying hens used in this period in commercial poultry farming. However, along with other native breeds, it was displaced from commercial use in the second half of the 20th century when improved foreign breeds and lines, which were much more productive, spread. The Utrerana breed was relegated from then on to backyard rearing and for its aesthetic values, having drastically reduced its census and worsened its laying performance as selection for productivity ceased. However, the relevance of the Utrerana chicken breed is such that conservation programs are being carried out and it was recently included in the Official Catalogue of Livestock Breeds of Spain. In this context, this article reviews the origin, selection, genetic characterisation and productive performance of the Utrerana chicken breed, evaluating the current status of this Spanish poultry genetic resource.

**Abstract:**

The conservation of threatened local livestock genetic resources involves characterising them to implement conservation strategies. The Utrerana is a Mediterranean-type chicken breed, included in the Official Catalogue of Livestock Breeds of Spain and in the Domestic Animal Diversity Information System (DAD-IS) of the Food and Agriculture Organization (FAO), native to south Spain created in 1926 by a farmer from Utrera (Seville province). It was selected for laying performance and with four plumage varieties (White, Black, Black-barred and Partridge), reaching average yields of 180 eggs per hen per year. It was widely used in commercial farming in the second quarter of the 20th century, being subsequently displaced in the second half of the 20th century by the spreading of the improved foreign breeds and lines. The Utrerana breed was reared from then on for its aesthetic values and in backyard systems, being endangered with a vulnerable local risk status (1822 birds in 2022 with an increasing trend) and having worsened its laying performance as selection for productivity ceased. The breed has received little attention from the research community. Therefore, this work aims to review the literature on the origin, selection, genetic and productive characterisation and status of the populations of the breed, as well as the conservation strategies. The Utrerana chicken is a polymorphic breed showing high genetic diversity, sexual dimorphism and morpho-functional differences among varieties. Currently, Utrerana hens start laying at 6 months of age, and a hen lays 94–121 high quality eggs (59–64 g) per year, showing seasonality. It is a slow-growing breed with mature weights of 2.4–2.6 kg for roosters and 1.9–2.0 g for hens. The Utrerana chicken breed is rustic and adapted to alternative farming systems. This review has identified research gaps to be filled, such as characterising the carcass and meat quality of the Utrerana chicken, and evidences the need to make efforts to promote the breed and expand its populations.

## 1. Introduction

There are 21 breeds of chickens (*Gallus gallus* Linnaeus, 1758) recognized as autochthonous breeds in the Official Catalogue of Livestock Breeds of Spain [1,2] and included in the Domestic Animal Diversity Information System (DAD-IS) of the Food and Agriculture Organization of the United Nations (FAO) [3]. One of these avian breeds is the Utrerana chicken [4,5,6,7], which presents interesting characteristics due to the way it was created and selected by a poultry farmer (Mr. Joaquín del Castillo [8]), due to the fact that it is a breed with four plumage varieties (Black, Partridge, Black-barred and White) [9], the relevance it reached in commercial poultry farming up to the mid-20th century [9,10] before being displaced by the more productive selected foreign commercial strains of laying hens [11,12], the way in which it has been preserved until today by amateur breeders [6,13,14] until it recently received official recognition [15], and being inextricably linked to the Andalusian town from which it originates and takes it its name (Utrera, province of Seville, southern Spain).

The Utrerana chicken was created almost a century ago [8] and is a light breed belonging to the Mediterranean group [16], with a notable aptitude for egg production [8,17]. It is reared under free-range, low-input conditions, has a rustic character [5,6] and displays a certain productive seasonality [18] due to the fact that the selection for egg laying that was undertaken in the second quarter of the 20th century [8] was later abandoned as it lost economic interest when improved, more productive hen strains spread in the second half of the 20th century [11,12]. After its abandonment as a breed used in professional poultry farming to produce eggs for consumption, it was kept by some amateur breeders who have maintained it to the present day mainly for its aesthetic values, selecting it only for morphological traits, and for the self-consumption of their products (mainly eggs and meat) [19,20].

As a consequence of this historical development, the Utrerana chicken breed, which became widespread throughout Spain and even abroad in its heyday [21], has now been relegated mainly to the region of Andalusia (southern Spain) and is nowadays endangered [22], although two different institutional programs have established recovery and conservation programs [20,23] and a group of amateur breeders, now integrated in the Asociación Nacional de Criadores de Gallinas Utreranas [24] (ANCGU; National Association of Utrerana Chicken Breeders, in English), promoted its recovery, its official recognition as an autochthonous breed and the creation of the Herd-book and the breed breeding program, supported by the Provincial Agricultural Centre of the Provincial Council of Cordoba, the Andalusian Institute of Agricultural and Fisheries Research and Training (IFAPA) and the Department of Genetics of the University of Cordoba [15,25,26].

In recent years the Utrerana chicken has been the subject of research, mainly for its genetic [27], reproductive (e.g., [28,29]) and functional and productive characterization [17]. However, a critical review is lacking that comprehensively analyses the state of the art regarding knowledge about this breed. Therefore, in this context, this work aims to review the origin, selection, genetic characterisation, productive potential and current situation of the Utrerana chicken breed, through the analysis of the scientific and informative bibliography published on the breed throughout its existence.

## 2. Methodology

A comprehensive literature search was conducted using PubMed, Scopus, Web of Science, Dialnet and TESEO databases, Google Scholar, the libraries of the University of Cordoba and the University of Seville (Spain), the website of the Official Catalogue of Breeds of Spain [7], the website of the Library of the National Institute for Agricultural and Food Research and Technology (INIA) [30], and the website and archives of the Asociación Nacional de Criadores de Gallinas Utreranas (ANCGU) [24].

The search included all relevant papers and publications found between 1926 (year of constitution and beginning of the selection of the breed) up to August 2023. It focussed on all scientific studies and informative publications that addressed all aspects related to the creation, selection, genetic characterisation, population status, conservation, husbandry and productive performance of the Utrerana chicken breed. A broad combination of search terms related to the Utrerana chicken breed was used, both in English and in Spanish, the following standing out: “Utrerana”, “Utrera”, “Andaluza Utrerana”, “avian breed”, “raza avícola”, “hen”, “chicken”, “gallina”, “Utrerana Franciscana”, “Andaluza Franciscana”, “Franciscan Utrerana”, “Andaluza Negra Barrada”, “Black-barred Andaluza”, “Utrerana Perdiz”, “Andaluza Perdiz”, “Black-Breasted Red Andaluza”, “Black-Red Andaluza”, “Utrerana Aperdizada”, “Partridge Utrerana”, “Utrerana Negra”, “Black Utrerana”, “Utrerana Blanca”, and “White Utrerana”.

This wide variety of search terms has been chosen because the Utrerana chicken breed is also known as “Andaluza” [4] (see Section 10.1 for an explanation), and has four varieties (named “Negra”, “Perdiz”, “Franciscana” and “Blanca” in Spanish). In addition, publications can be found with the respective denominations in Spanish or English, which generates heterogeneity in the naming of the breed and its varieties in the literature. As the breed was officially recognized under the name of Utrerana [15], in this review this name has been chosen for the breed in most of the paper, except in Section 10.1, in which the denomination “Andaluza” has also been used to refer to the birds of the conservation program maintained by the National Institute for Agricultural and Food Research and Technology (INIA). For the varieties, the following English names were chosen: “Black”, “Partridge”, “Black-barred” and “White”, as a translation of Spanish names “Negra”, “Perdiz”, “Franciscana” and “Blanca”, respectively.

The review has also been given a historiographic approach for a better understanding of the evolution and current status of the breed.

## 3. The Beginnings: Constitution and Selection of the Utrerana Chicken Breed

An invaluable document for understanding how the Utrerana breed emerged is the book published by its creator, Mr. Joaquín del Castillo [8], entitled “The Utrerana hens. History and description of this new breed” (“Las gallinas Utreranas. Historia y descripción de esta nueva raza”, in Spanish) and edited by Ediciones Tipografía Moderna, from the town of Utrera (Seville, Spain). Composed of 135 pages, plus five colour plates (including those of the ideal type of the four plumage varieties of the breed), a map and nine graphs, this bibliographic gem, well known among breeders and lovers of this chicken breed, tells firsthand the breed selection process from a founding animal stock made up of unselected hens with good potential for laying large eggs collected from ranches and farmhouses in the Utrera countryside and surroundings. In the area, hens of large body size were usually reared by choosing the biggest roosters to mate them, and eggs were selected for their big size. Birds were reared under a free-range regime with feeding based almost exclusively on the natural resources of the country with little supplementation of grain. These birds were rustics and with a late maturity, beginning to lay at an age of at least 10 months. The feather colour was not fixed and there were birds of all colours [8].

The Utrerana breed selection was carried out at the Santa Matilde Farm, where Mr. Joaquín del Castillo poultry farmer established an egg production farm in 1926. This labour began with the acquisition of eggs weighing 80 g on average, with a white eggshell, which were subsequently incubated and produced nearly 400 chicks reared without artificial heating. From these chicks, 200 hens and several roosters of varied feather colours were chosen to serve as breeding birds and submitted to laying control. During the first laying year, the average egg yield per hen was 111 eggs, varying between 53 and 189 [8]. Mass selection was subsequently used to fix and improve the breed, initially only for production performance, by using trap nests and trying to eliminate broodiness. In this initial period, selection was carried out by forming a single breeding flock each year with the best laying hens from the previous year, which were mated with roosters born from the best-performing hens from the previous year. The number of laying hens in the 1926–1927 to 1930–1931 campaigns was 141, 248, 380, 548 and 691, respectively. After the first five years of selection, an average of 187 eggs per hen and year was reached, illustrating the huge genetic progress achieved. During this initial period, no selection of the exterior of birds was carried out [8].

In order to achieve uniformity of colour and conformation of the birds, the next step undertaken by the founder of the breed was the selection and consolidation of the four plumage varieties: White, Black-barred, Black and Partridge. This task started in 1931 by means of lines selection, establishing at least two lines for each variety [8]. The colour of the plumage of the White variety is due to the allele *I* (dominant white, melanin inhibitor), that of the Black-barred is due to the alleles *E* (black) and *B* (sex-linked barred), that of the Black is due to the allele *E*, and that of the Partridge is due to the *e*^+^ allele of the multiallelic series *E*/*e*, which gives the primitive or wild phenotype of the native species red junglefowl (*Gallus gallus bankiva* Linnaeus, 1758) [4,31]. Initially, the Black, White and Black-barred varieties were selected and fixed, and later the Partridge variety was extracted from the Black [9], being still in selection in 1950 [21]. The focus of the selection on the exterior traits initially implied a slowdown in the genetic progress of the average egg yield, although it was possible to maintain it around 180 eggs per hen per year [8].

This book includes the breed standards of the four varieties of the breed (see Section 5), and cites the first recognition of each variety in various Spanish poultry shows of the time: the White in 1946 (National Poultry Exhibition in Barcelona), the Black-barred in 1948 (National Exhibition and Assembly of Poultry Farmers in Madrid) and the Black and Partridge in 1949 (National Poultry Exhibition in Seville) [8].

In addition, this book describes the various zootechnical aspects of the production, husbandry and management of this breed in Santa Matilde poultry farm, such as housing, feeding, hygiene practices, reproduction, artificial incubation and rearing of chickens. It also reports that the eggs were of good size, with average weights of 62–64 g and of white or off-white shell colour [8].

Three criteria were used to select the best-performing hens [8]: (i) *autumn laying*: number or eggs laid per hen during October, November and December, an interesting measurement because this is the period of the year with lower production; (ii) *total laying*: number of eggs laid per hen during the first 19 months of life, which corresponds to a year of production due to the fact that the hens began their productive life at an average age of 6.5 months; and (iii) *egg weight*: calculated (in grams) by averaging the weight of one egg per hen and per month between June and November. With these three measurements, the selection index for each hen was calculated as a score obtained by the sum of *total laying* and *autumn laying*, and then this value was multiplied by *egg weight*. This number was divided per 1000 and kept with two decimal places. It is equivalent to the egg mass laid per hen and year in kilograms, but by weighting the eggs laid in autumn twice, it was possible to select hens that laid many eggs of high weight per year, and that laid a relevant number of eggs in autumn.

The book by Mr. Joaquín del Castillo [8] also attests to the genetic progress achieved by fixing and improving the breed in terms of egg laying performance, since it refers to an initial production of 111 eggs per hen per year at the beginning in the foundational animal base and that, after the improvement work, average productions of 180 eggs per hen were achieved in barely 25 years between the beginning of the work in 1926 and the publication of the book in 1951. This allowed the new Utrerana breed to reach rapid and wide dispersion (see Section 6) and to be placed at the level of other good breeds of laying hens used in poultry production at the time, such as the Black Castellana (the most widespread and productive Spanish autochthonous breed at the time [32]) and White Leghorn (a foreign breed selected in the United States of America that was already spreading throughout Spain and that dominated intensive laying poultry farming thereafter [12]). In fact, in the egg laying contests at which the Utrerana breed participated between 1939 and 1948, the records reached were 177–239 eggs/year for White, 170–227 eggs/year for Black-barred, and 177–216 eggs/year for Black Utrerana varieties [8].

## 4. The First Report on the Utrerana Chicken Breed in a Scientific Journal

The publication in 1951 of a paper [21] in the prestigious scientific journal World’s Poultry Science Journal, the organ of scientific diffusion of the World Poultry Science Association (WPSA) which is still in publication today, is a relevant example of the evidence of the rapid consolidation and dispersion of the Utrerana chicken and the significant interest in it even outside national borders. Said article, entitled “Spanish breeds of chickens: The origin of the Utrera breed” [21], essentially transcribes some information contained in the aforementioned book by Mr. Joaquín del Castillo [8], mainly related to the description of the initial constitution of the breed and its selection at the “Santa Matilde” poultry farm, and the description of the four plumage varieties. It also includes the breed standards of the White Utrerana and the Black-barred Utrerana varieties.

## 5. Publication of the Breed Standards of the Varieties of the Utrerana Chicken Breed

Another early testimony of the consolidation of the Utrerana chicken as a breed is the publication of the breed standards of the four plumage varieties in the Spanish Book of Poultry Standards (“Libro español de patrones avícolas”, in Spanish) [9], published in Barcelona (Spain) by the extinct Association of Spanish Breeders of Select Birds (“Agrupación de Criadores Españoles de Aves Selectas”, ACEAS, in Spanish). Apart from the interest in itself that the recognition and dissemination of breed standards implies, the pictures of the roosters and hens of the four plumage varieties included in the book stand out as a valuable graphic testimony, since they attest to the conformation of the breed in its heyday. In addition, this book also informs about the geographic dispersion reached by the breed (see Section 6).

The breed standards in force for the Utrerana chicken breed, which are shown in Table 1, Table 2 and Table 3, correspond to the historical ones proposed by Mr. Joaquín del Castillo [8] and published by the Agrupación de Criadores Españoles de Aves Selectas [9], since these were approved by the resolution of 4 December 2013, of the General Directorate of Agricultural and Livestock Production of the Regional Government of Andalusia [33] after the official recognition of the breed (see Section 15).

To distinguish the birds of the Utrerana breed from birds of other similar breeds, their adjustment to the breed standard is evaluated paying special attention to the colour of the plumage, beak, legs, toes and nails, as well as to conformation proportionality. Furthermore, the inclination of the back must be 45°, the hen the comb must fall to one side of the head, covering one eye, and the hen the tail must be shaped as an axe blade.

## 6. Evolution and Current Situation of the Population of the Utrerana Chicken Breed

The knowledge about the geographic distribution and the numerical abundance of the populations of the Utrerana chicken breed has been variable throughout its history, and it is difficult to reliably estimate them due to the absence of specific censuses. Reports on its distribution and abundance are summarised here.

The founder of the breed, Mr. Joaquín del Castillo [8], reported in 1951 that the rusticity and vigour of the Utrerana chickens made them suitable to acclimatize in the most extreme climates and to accommodate very different farming or rearing regimes, obtaining from them excellent results both in intensive farms and in peasant farmyards or pens, as well as under full free range. At that time, the breed prospered in various climates of the Spanish territory, both in warm areas of Andalusia and the Canary Islands, and abroad in Morocco and the Gulf of Guinea, as well as in the temperate Balearic Islands and the Levante coast, or in the colder areas like Aragon, both Castile regions or, finally, the humid provinces of the Cantabrian area and Galicia. He points out that from 1934, the year in which it began to be marketed, until 1951, the breed spread in all the Spanish provinces and in the then Spanish territories of Africa. Most of the hens of the breed were found in the Seville and neighbouring provinces, where most of the birds kept on modern farms and a large proportion of free-range ones were of the Utrerana breed.

Liaño et al. [9] confirmed that in 1953 the Utrerana breed was almost always bred in large groups in all the Spanish peninsular provinces and in the Balearic Islands, as well as in Morocco and in the Gulf of Guinea, noting that it had reached its greatest dispersion in Andalusia, where it had become the main breed in poultry production. A few years before, Rabanal [10] reported that the most used breeds of laying hens in Spain were the White Leghorn, Black Castellana, Prat Leonada, Rhode Island Red and the Utrerana (White, Black and Black-barred varieties), the latter predominantly in Andalusia, the Balearic Islands, the Canary Islands and Morocco. In 1951, Piedrafita [21] reported that the expansion in large and small flocks was greater every day, and estimated that a million and half of Utrerana chicks were produced every year among all poultry establishments. Orozco [4] confirms that this breed was broadly used as a productive laying hen during the 1940s and 1950s. Cárceles [34,35] classified the Utrerana among the national productive or modern breeds, which were used extensively at the beginning of the development of industrial poultry farming in Spain as high-quality layers.

However, commercial farming of the Utrerana breed was later abandoned as it lost economic interest when improved, more productive hen strains spread in the second half of the 20th century in Spain [11,12]. Thus, in the opinion of Orozco and Campo [23], published in 1981, the Utrerana chickens had been completely lost by the 1980s, indicating that they had verified this when trying to find them for the INIA’s conservation program (see Section 10.1), not only in the surroundings of Utrera but also in various points in the province of Seville. They also indicate that they had verified the existence of Mediterranean-type birds in various Andalusian farmhouses, which, being mixed, presented in segregation the colours of the Utrerana chickens. Cárceles [34,35] considers that the Utrerana breed could still be found in 1983 in the Andalusian countryside but it was almost extinct.

Rodero et al. [19], in visits carried out at the beginning of the 1990s to Andalusian farms to prospect for animals of autochthonous breeds of other species, report having found hens that derived from the Utrerana breed but were crossed with other foreign breeds. These were animals with similar characteristics to the Black Utrerana, small in size, which frequently interbred with the White Leghorn by absorption. Rodero et al. [19] indicate that these birds were raised extensively in farmyards in small numbers, for the owner’s self-sufficiency in eggs and meat. At that time (1994) these authors did not report any chicken conservation program in Andalusia.

Lancho [36] estimates that in 1998 there was a census of about 800 Utrerana chickens, with a predominance of the Morisca and Partridge varieties, and the Black-barred and Black varieties being found in fewer numbers.

León and Cabello [5] report that the situation of the populations of the breed in 2009 was in clear decline, indicating that it was also largely motivated by the uncertainty that avian influenza outbreaks was causing among breeders.

Garcia-Romero et al. [6] mention that the breed did not become extinct in recent times thanks to the work of amateur breeders such as Mr. Juan Manuel Sánchez Ocaña. The estimated census in 2018 was 300–500 birds, distributed mainly in the provinces of Seville, Cadiz, Málaga and Cordoba, with some small nuclei in the Castile–La Mancha region.

In more recent times, the only reliable information on censuses of the Utrerana chicken breed is that related to the birds registered in the Herd-Book (see Section 18).

Several circumstances have contributed to the weak situation of the Utrerana chicken populations in the last decades, such as the strength of the intensive sector of poultry meat and egg production that marginalised traditional production based on local breeds [37], and even the delay of the FAO in including avian breeds in their conservation programs for livestock genetic resources, a situation that began to be reversed when various programs and projects for the recovery of the breed were addressed in Spain [20,37] (see Section 10).

## 7. Current Husbandry of the Utrerana Chicken

As indicated in Section 6, once the Utrerana chicken stopped being used in intensive poultry production systems, it went back to being used exclusively in farmyards and free-range systems in small numbers, for the owner’s self-sufficiency in eggs and meat, as well as for its aesthetic values [19], in a similar way to how hens from the original populations from which the breed was selected were raised [8]. The current breeding system and husbandry of the Utrerana chicken [5,6] is characterised by the fact that the birds are rustic and very strong, showing high resistance to infectious-contagious and parasitic diseases and low mortality. Birds are resistant to summer heat and winter cold and have the ability to run, fly to some extent, graze, and peck, being adapted to dryland agrosystems and to free-range and extensive organic farming, also with outdoor housing. Birds feed based on grazing supplemented with cereal grains and legumes, or with balanced feed, consuming many invertebrates. Productive life and longevity last between 3 and 6 years, with puberty reached at 180–200 days of age. The breed it is reported to have good laying performance yielding 150–180 eggs per hen per year (120–180 according to Campo [31])—a value that could be overestimated, as can be seen in Section 12—with good fertility (90%), and lays white or off-white eggs weighing 55–80 g; in general, it does not show broodiness. The ideal rooster:hen ratio is 1:4. The growth of the roosters is completed in just over a year and that of the hens in six months.

## 8. Early Use of the Utrerana Chicken Breed in Scientific Research

An example of evidence of the relevance achieved by the Utrerana chicken breed shortly after its creation and expansion, and once it spread in poultry production, was its use in scientific research at the time, as were other autochthonous and improved foreign breeds. An example of scientific research that used the Utrerana chicken breed as an experimental animal is the study on vitamin T carried out by Torres Marty and Vall Bañeres [38], in which this breed was used as an animal model together with mouse. In this trial, chickens from the cross between roosters of the Black Utrerana and hens of the Black-barred Utrerana varieties were used, which allows knowing the sex at birth, and found that the chickens fed with the addition of vitamin T reached higher weight, average daily gain and uniformity of development after three weeks. Another example of the use of the Utrerana chicken breed in scientific research is the study on the erythrocyte sedimentation rate in birds carried out by Medina Blanco and Gómez Cárdenas [39]. In this trial, Utrerana birds of the White and Black-barred varieties were used, together with birds of the Spanish autochthonous breeds Black Castellana, Prat and Carmelitana, in addition to the White Leghorn breed, and reported diseases that increased the rate of erythrocyte sedimentation.

## 9. Initial Genetic Characterisation of Utrerana Chicken Breed

The first scientific investigations undertaken on the genetic characterisation of Utrerana chicken breed in the university environment were carried out by Dr. Antonio Rodero, Professor of Genetics at the Faculty of Veterinary Medicine of the University of Cordoba (Spain). These investigations were carried out at the beginning of the 1960s and were published in the journal Archivos de Zootecnia [40,41,42]. This research focused on the Black-barred Utrerana variety, which was compared in several trials with the Black Castellana and White Leghorn breeds. These are the first studies on the heritability of weight at different ages carried out on Spanish chicken breeds and dealt with three aspects.

In the first of these studies, the heritability of the live weight of birds of the three aforementioned breeds was studied, by using birds belonging to a commercial farm in the province of Cordoba [40]. It reported that the mass or individual selection was suitable to improve the live weight of hens at four months of age. It was also found that the heritability of live weight was high, and higher in the Black-barred Utrerana hen (*h*^2^ = 0.54) than in the White Leghorn and Black Castellana breeds (*h*^2^ = 0.35 and 0.34, respectively). Maternal effects accounted for 14% of the variability in live weight of Black-barred Utrerana variety. Of the three avian breeds under study, the Black-barred Utrerana is the one that had undergone the least selection, and therefore it is possible that it had more variability in its genotype, which produces a higher heritability. This research reveals the good potential of the Black-barred variety of the Utrerana breed to improve its live weight and growth (see Section 13 for varieties growth performance).

The second of these studies focused on investigating the influence of the breed and the rooster on some traits of interest [41]. Thus, it was confirmed that the live weight of the birds at four months of age was higher in the Black-barred Utrerana variety (1.469 kg), followed by the Black Castellana (1.408 kg) and the White Leghorn (1.375 kg), in descending order. It was also determined that the rooster influences the fertility of the hens with which it is mated, while it does not influence the hatchability of the eggs or sex ratio of the offspring at three month of age. The fertility and hatchability of the total eggs was found to be higher in the Black Castellana (92.8% and 80.8%, respectively) than in the Black-barred Utrerana (87.8% and 73.5%) and the White Leghorn (90.3% and 77.4%) breeds, which both showed similar fertility and hatchability.

The third study analysed, in the three aforementioned breeds, the genetic correlations among various traits, such as fertility, hatchability, mortality, resistance (index obtained subtracting, to live birds, mortality plus culling), sex ratio and live weight at four months of age, determined for hens [42]. In summary, for the Black-barred Utrerana variety, significant correlations were only found between (i) fertility and hatchability (r = 0.838), fertility and sex ratio (r = −0.505) and fertility and resistance (r = −0.952); (ii) hatchability and sex ratio (r = −0.456) and hatchability and resistance (r = −0.806); (iii) mortality and resistance (r = −0.448); and (iv) sex ratio and resistance (r = 0.520).

## 10. Conservation Programs for the Utrerana Chicken Breed

As already mentioned above, after being displaced from commercial poultry farming in the 1950s and 1960s due to the expansion of hybrids to produce eggs and meat in the industrial poultry farming, the Utrerana breed experienced a population decline that led it to be in danger of extinction by the 1970s (see Section 6). This aroused interest in attempting to recover and preserve the breed [23,36]. Thus, throughout its existence, the Utrerana chicken breed has been the subject of two ex situ genetic conservation programs, which continue to operate today and have contributed not only to conserving the breed but also to research on it. These programs are described below.

### 10.1. Program for the Conservation of Spanish Chicken Breeds of the National Institute for Agricultural and Food Research and Technology

The Department of Animal Genetic Improvement of the National Institute for Agricultural and Food Research and Technology (INIA, “Instituto Nacional de Investigación y Tecnología Agraria y Alimentaria”, in Spanish) founded the Program for the Conservation of Spanish Chicken Breeds in 1975 [23]. In this program, which is still operating today, ex situ conservation closed nuclei of 12 native Spanish breeds of chickens are maintained in El Encín Research Station (Madrid, Spain). Since these breeds were being replaced by the more productive commercial hybrids, the objectives of this program are the genetic study and conservation of genetic resources of Spanish breeds of chickens, as well as the dissemination of this genetic material among breeders interested in productive systems with high resilience animals adapted to the environmental conditions of the Iberian Peninsula. Regarding the Utrerana chicken breed, this conservation program currently maintains two nuclei of the Partridge and Black-barred varieties of the breed, which in this program are named “Andaluza Perdiz” (“Black-Breasted Red Andaluza” in English) and “Andaluza Negra Barrada” (“Andaluza Franciscana” or “Black-Barred Andaluza” in English”), respectively [43]. The reason behind the fact that the varieties maintained in this nucleus are called “Andaluza” more frequently than “Utrerana” is due to the fact that when the nuclei were formed the populations from which birds were obtained were of diverse origin (not only from Utrera), because these birds do not come from the same Andalusian chickens that proliferated in the old Spanish poultry farming industry and because the chickens from the El Encín nuclei present some colour variants that make them different in a certain way [4].

In the nuclei of this program, the hens are reared in henhouses, with trap nests, and in a ratio of one rooster for every five hens, with balanced mating that implies a rotational change of males, and are maintained in adequate effective sizes (ranging from 100 to 300 for different breeds) [44].

There were three initial objectives of the INIA Program: (i) location of birds, (ii) organization of the conservation scheme, and (iii) genetic study of quantitative and qualitative characters [45]. When the initial nuclei of Utrerana or Andaluza chickens were established at the El Encín Research Station, flocks of the Black, Black-barred and Partridge varieties began to be formed [23]. The original Utrerana chicken had completely disappeared, but birds included in more or less heterogeneous populations were found in the provinces of Cordoba, Seville and Badajoz [45]. Orozco [4] reports in detail from which breeders and farmhouses the first birds were gathered. In 1981 there were 30–40 birds of the Black variety, 40–50 birds of the Black-barred variety and 10–20 birds of the Partridge variety in the INIA’s nuclei, this last variety being the scarcest and the most difficult to find in the countryside [23]. In 1982, the nuclei were composed of 100 hens and 20 roosters of the Black variety, 40 hens and 10 roosters of the Black-barred variety, and 10 hens and 3 roosters of the Partridge variety, all of them being yet to be purified and presenting undesirable segregations [45]. By 1981, more birds of the breed were being searched for in Andalusian farmhouses to dilute the consanguinity [23]. At that time, the INIA’s conservation program was working mainly on the genetics of the colour of the plumage of the breeds. In 1985, ten years after this conservation program was founded [46], the flock size was 50 birds of each of the Black-barred and Partridge varieties and about 100 birds of the Black variety. The Black variety presented some segregation in the colour of the legs and the degree of eumelanisation of the Black-barred variety was not optimal, especially in the down of the newly hatched chick, something essential for the expression of the white occipital spot produced by the sex-linked barred gene. In the Partridge variety, the presence of the *e*^+^ allele seemed to have stabilised, after having eliminated undesirable segregations both for the remaining alleles of this locus and for those of the Columbian type. Orozco [4] and Campo [31] summarise the genetics of plumage, skin and leg colour of varieties maintained at the El Encín Research Station. In 2005 [31] there were about 12 roosters and 60 hens in pedigree pens and 30 roosters and 125 hens in normal pens. The effective population size was Ne = 164, equivalent to an increase in inbreeding of 0.3% per generation and a situation of only potential danger.

Orozco [4] explains that there was no interest in recreating the White variety at the El Encín Research Station because it was considered that the white colour was not often found in native hens and because of suspicions that the White Leghorn had an intervention in this variety.

In addition to research more directly related to the conservation and genetic characterisation of the Andaluza breed (see Section 11), research has also been carried out in other fields of knowledge within the framework of the INIA’s Program for the Conservation of Spanish Chicken Breeds, using birds from the nuclei of the El Encín Research Station. Most of these studies have been carried out on reproduction and artificial insemination, as well as on animal welfare. The main studies carried out within the framework of this program (also jointly with INIA’s Department of Animal Reproduction) using birds of the Andaluza breed are summarised below. Part of this research is related to semen cryopreservation because a germplasm bank based on semen cryopreservation was also created, which complemented the in vivo INIA’s conservation program as a way to preserve the autochthonous breeds from sanitary risks, such as avian influenza [47,48,49].

Using birds of the Black-barred variety, Abouelezz et al. [50] studied the contraceptive effects of glycerol and egg yolk when used as sperm cryoprotectants in artificial insemination. Given their known inhibitory effects on the fertilization of ova in birds, they found that the maximum concentrations tolerated by the rooster frozen-thawed sperm are 0.75% for glycerol and 7.5% for egg yolk. The contraceptive actions of glycerol and egg yolk are exerted through different ways; thus, high glycerol concentrations reduce fresh rooster sperm penetration counts and fertilising ability, therefore affecting the transit of sperm through the oviduct to the infundibulum area, while the contraceptive mechanism of egg yolk remains unclear. Prior to this study, Santiago-Moreno et al. [51] verified with pooled semen of 16 breeds (including Black-barred and Partridge varieties) used for artificial insemination on hens of the Black-barred variety, Black Castellana and Red-Barred Vasca breeds, that the addition of chicken or quail egg yolk protects rooster sperm cells against cold shock and during freezing and thawing but exerts a contraceptive effect in the genital tract of the hen. Pérez-Garnelo et al. [52] compared the effects on seminal parameters of glycerol and dimethylacetamide (DMA) as cryoprotectants in the freezing of rooster semen of several breeds (Black-barred and Black Andaluza, Black Castellana, Penedesenca Negra and Prat Leonada), using straws and pellets for DMA and straws for glycerol. After thawing, the cryoprotectant exerted a significant effect on the seminal parameters, and the breed affected membrane integrity and percentage of normal sperms (lower and higher values in Penedesenca Negra, respectively, and no difference between Black-barred and Partridge varieties). Moreover, a sperm penetration test showed that the potential fertility of the semen doses was higher for all breeds when DMA was used as cryoprotectant when freezing was carried out in pellets, except for the Black-barred variety, for which better results were achieved when DMA and straws were used.

A research work carried out by a team of researchers from INIA and the Faculty of Veterinary Medicine of the Complutense University of Madrid (Spain) has investigated the influence of breed and photoperiod on the sperm quality of roosters of the Black Castellana, Prat Leonada, Penedesenca Negra and Black-barred and Partridge varieties of the Andaluza breed [53,54]. It has been confirmed that sperm production is seasonal, a typical characteristic of poorly selected autochthonous breeds, and that it is of better quality with increasing photoperiod, in correspondence with the fact that the *Gallus gallus* species typically reproduces optimally with long days, and that the photoperiod effect is more marked in some breeds than in others [54]. Anguita et al. [53] and Pérez-Garnelo et al. [54] also note that sperm concentration (sperm/mL) and total ejaculate sperm count are affected by breed and photoperiod, while ejaculate volume depends only on breed. Comparing the aforementioned breeds, they confirm that sperm production is higher in the Black-barred variety, followed by the Prat Leonada; then there is the Partridge variety, the Penedesenca Negra and, finally, the Black Castellana breed. Regarding the qualitative parameters of the ejaculates, such as sperm motility and quality, Anguita et al. [53] and Pérez-Garnelo et al. [54] confirm that they are influenced by both the photoperiod and the breed, so that the quality of the ejaculates is higher in the Black-barred variety, followed by the Prat Leonada, the Black Castellana and the Penedesenca Negra (all three breeds at the same level) and, lastly, the Partridge variety.

Research has also been carried out on the influence of seminal plasma, which modulates sperm function in the reproduction process, on sperm cryopreservation in Spanish chicken breeds, including Partridge and Black-barred varieties [55,56]. No differences in the viability of fresh sperm or freezing–thawing response of samples without seminal plasma among breeds were found. However, sperm samples with seminal plasma showed differences depending on the breed, with the Partridge variety displaying the lowest sperm viability and the Black-barred variety being intermediate [55,56]. Santiago-Moreno et al. [56] demonstrated that freezing–thawing with seminal plasma is influenced by the breed, increases DNA fragmentation, and specific amino acids in seminal plasma are linked to DNA integrity.

The effect of season on the semen freezeability of free-range roosters from 14 Spanish chicken breeds, including Black-barred and Partridge Andaluza varieties, was assessed by Santiago-Moreno et al. [28] in samples frozen in straws using DMA as a cryoprotectant. No seasonal effects were reported on fresh semen quality, neither the percentage of viable frozen–thawed spermatozoa nor the percentage with an intact acrosome. However, spring was reported to be the best season to collect and freeze semen of the Spanish chicken breeds under free-range rearing because the percentage of immotile spermatozoa was lower in this season than in summer or autumn; the percentage of spermatozoa showing progressive motility, and curvilinear velocity, straight-line velocity and average path velocity values of sperm were higher in spring than during the rest of the year.

Santiago-Moreno et al. [57] investigated the sperm velocity associated with chicken breed in last male precedence, by means of a trial in which Andaluza Azul and Black Castellana hens mated for three weeks with roosters of other breed were subsequently mated for three additional weeks with Black-barred Andaluza roosters. The plumage of the chicks reveals the breed whose sperm has fertilised the eggs. Because it was the second rooster breed replacing the first, Black-barred variety sperm fertilised the majority of eggs in both cases, and the percentage of chicks obtained from Andaluza Azul was higher than that of chicks obtained from Black Castellana when these breeds were used as the first rooster to mate hens, while for the hens fertility the opposite was observed. The sperm of Black Castellana, Black-barred variety and Andaluza Azul are considered of good, medium and low quality, respectively, and the results of this trial showed that sperm of the Andaluza Azul breed compensates its moderate fertility via better sperm movement, thus being advantageous when they are involved in competition with roosters of other breeds.

The effects of the presence of hens on the sperm variables of roosters have been investigated using the Black-barred variety. It has been found that the presence of hens with roosters, when hens were introduced to the roosters when the latter were 22 weeks old, does not modify the sperm quantitative variables (volume, number of spermatozoa per ejaculate and sperm concentration) at 36 weeks of age, but it decreases the percentages of non-progressive motile sperms and slow sperms, as well as increases sperm velocity (both straight line and curvilinear velocities), which can improve semen fertility and could be of interest because optimal sperm motility is a criterion used in the selection of sperm donor roosters [58,59].

Among the investigations related to animal welfare carried out with birds of the Andaluza breed from the nuclei of the El Encín Research Station, the following stand out. Considering that the chicken is the only domestic species that was initially selected for aggressiveness instead of docility, Dávila et al. [60] analysed the relationship between aggressiveness and the ratio between heterophils and lymphocytes (H/L; an indicator of stress) or the duration of tonic immobility (a measure of fear) in roosters of different breeds, including the Partridge and Black-barred varieties of the Andaluza breed. It was found that that the roosters of these varieties of the Andaluza breed showed a relative aggressiveness that was intermediate compared to that of other Spanish autochthonous breeds, with no difference between both varieties. The roosters of the Black-barred variety and Cara Blanca breed had the maximum duration of tonic immobility and those of the Leonesa breed had the minimum, with the Partridge variety showing intermediate values (but with no difference with the Black-barred variety). There were no significant differences in the leukocyte ratio among breeds. These results suggest that genetic selection for egg laying could have contributed to the moderate aggressiveness of the Andaluza breed. Gil et al. [61] investigated the hetreophil to lymphocyte ratio response of 10 Spanish autochthonous breeds of laying hens to cold and heat stress and water restriction, as a way to assess their ability to cope with environmental challenges arising from climate change that affect productive performance. Based on their H/L response, the Partridge variety was among the breeds with lower overall resilience to stress, while the Black-barred variety was intermediate. Both varieties showed similar response to cold stress and water restriction, while the Partridge variety coped worse with heat stress than the Black-barred variety do.

Santiago-Moreno et al. [62] assessed the influence of access to pasture in an outdoor housing system on rooster sperm quality and response to cryopreservation, as well as its correlation with welfare indicators, by using birds of the Andaluza Black-barred variety and Red-barred Vasca breed. Ejaculates from roosters with access to pasture showed higher percentages of sperm progressive motility and a higher motility index but, unexpectedly, these birds showed a higher H/L ratio. No effect of the access to pasture was found on semen quality after freezing–thawing, excepting for the Red-barred Vasca breed roosters with access to pasture, whose semen showed a higher percentage of progressive motility when compared to roosters of the same breed with no access to pasture. Moreover, correlations were found between H/L ratio and sperm motility, progressive motility and sperm motility index. No effect of the access to pasture was reported on the duration of tonic immobility. Moreover, sperm appearance, motility index and sperm concentration of fresh samples, as well as sperm motility in frozen-thawed samples, were higher in the Black-barred variety than in the Red-barred Vasca breed, independently of the access to pasture regime. It was concluded that access to pasture improves fresh sperm motility.

Using chicks from a F1 cross between Black-barred variety and Black Castellana breed, it has been reported that their rearing with or without a broody hen does not affect their stress [63]. Chicks reared without a broody hen showed longer tonic immobility duration than those reared with a broody hen, suggesting that its presence reduces fear in chicks, while there was no effect of the presence of a broody hen on H/L ratio and the relative fluctuating asymmetry of several anatomic parts of the limbs.

### 10.2. Andalusian Conservation Programs of the Utrerana Chicken of the Provincial Agricultural Centre of the Provincial Council of Cordoba, the Department of Genetics of the Faculty of Veterinary Medicine of the University of Cordoba and the Andalusian Institute of Agricultural and Fisheries Research and Training (IFAPA)

In Andalusia, the region of origin of the Utrerana breed, despite original populations having survived in rural areas, these have reached the present in conditions of total oblivion and without connection among them. In many cases, their continuity over time has been ensured more for their aesthetic values or for the emotional reasons of the breeders than for their productive potential [20]. This is an anomalous situation, since the studies carried out to establish the breed [8] revealed that it had great aptitude for laying eggs, a fact that has not been rigorously investigated until recently [20], nor had recovery programs been established in the region. In Andalusia, recovery and conservation programs for this avian breed were implemented relatively recently, under a set of initiatives and collaborations between three institutions (Provincial Agricultural Centre of the Provincial Council of Cordoba, Department of Genetics of the Faculty of Veterinary Medicine of the University of Cordoba and IFAPA Centre of Cordoba), having led to the creation of an ex situ conservation centre and the completion of several investigations, which are described here.

Lancho [36] describes a relatively recent first analysis of the situation of the native poultry breeds of Andalusia and the need to conserve them, and mentions that already in 1998 there was a group of poultry breeders from Utrera collaborating for this purpose with the Department of Genetics of the Faculty of Veterinary Medicine of the University of Cordoba. Lancho [36] reports that work was being conducted on a project on “Recovery of the autochthonous breeds of Andalusian chickens” financed by the Department of Agriculture and Fisheries of the Regional Government of Andalusia, which had the following objectives: (i) visit the relevant nuclei of chickens and create a database with the information collected, as well as introduce it into animal genetic resource banks (such as DAD-IS, FAO, where available information on the Utrerana breed is not currently complete); (ii) review the description of the breeds in traditional poultry farming treatises, and carry out a modern ethnological characterisation based on quantitative and qualitative variables; (iii) acquire adult birds and/or hatching eggs to create ex situ nuclei with flocks of 50 hens and 10 roosters of each breed, on which to carry out pedigree controls and production records; (iv) mating of the flocks of said nucleus will be planned to maintain genetic variability, minimising the increase in inbreeding in successive generations (using the coancestry matrix) and selecting the breeding candidates according to the proportion of founding animals in their pedigree (using the genetic conservation index); the descendants will be subjected to morphological mass selection and later to productive selection; (v) chicks will be delivered to new breeders to increase the effective and real size of the population, as well as to diversify the nuclei, and to stimulate the increase in genetic variability, the own flocks will be integrated with the existing nuclei and with the new ones, in a circuit of circular use, using the processes of drift and controlled genetic migration; and (vi) with the knowledge obtained in this pilot project, there will be a basis for proposing measures to develop a program for the reintroduction of native poultry flocks in Andalusia. Three years later, Lancho et al. [64] described the situation of this project, reporting that the ex situ conservation centre had been located in the Provincial Agricultural Centre of the Provincial Council of Córdoba since 1998. At that time, the censuses of the varieties of the Utrerana chicken breed in said conservation centre were 3 roosters and 15 hens of the Partridge variety and 1 rooster and 6 hens of the Black-barred variety. Moreover, the existence of few pure nuclei of these breeds in the countryside was highlighted, with birds that presented high levels of consanguinity. As successive steps of this program, two were mentioned: (i) to continue looking for nuclei of the breed in rural areas to increase the genetic variability of the base population; and (ii) to implement a program for the transfer of breeding stock to poultry breeders with the objective of stabilising and increasing the censuses, and thus achieving a productive orientation of these animals that makes them competitive in alternative poultry farming systems.

Magallanes et al. [65] describe results from a project led by the IFAPA Centre of Cordoba, pointing out that, in addition to birds gathered from Andalusian breeders, in the conservation centre established in the Provincial Agricultural Centre of the Provincial Council of Cordoba with the participation of the IFAPA Centre of Cordoba and the Department of Genetics of the University of Cordoba, there were also incorporated birds of the Partridge and Black-barred varieties of the Utrerana breed from the INIA’s nuclei at the El Encín Research Station. In 2004, the number of founding birds in the Andalusian nucleus were 6 roosters and 10 hens of the Black-barred variety and 11 roosters and 3 hens of the Partridge variety. During 2000–2001, research lines were opened, aimed at giving birds of the breed a commercial outlet by improving reproductive and productive traits. During 2001–2002, the laying performance of the founder nuclei was evaluated with a view to genetic improvement by production performance. Thus, it was reported that the hens of the Partridge variety from this nucleus laid eggs with a lower weight, width and length than the Black-barred variety hens (55.6 vs. 64.9 g, 41.5 vs. 43.3 mm, and 56.7 vs. 61.7 mm, respectively). The Partridge variety laid eggs with a greater range and higher maximum weight than the Black-barred variety. In addition, an analysis of covariance introducing the month of egg laying in the model to eliminate temporal effects revealed significant interbreed differences for egg weight and dimensions, allowing inferring a functional specialisation of the breed. During 2002–2003, works on the meat production of the breed began, obtaining growth curves and calculating the feed conversion ratio, as well as evaluating the speed and growth aptitude of both sexes. This information was intended to be used to select egg production animals that exceed minimum growth values. It was also intended to evaluate the meat aptitude of the breed to obtain capons.

Thus, since 1998, the Agricultural Provincial Centre of the Provincial Council of Cordoba has been developing programs for the genetic improvement and conservation of Andalusian poultry breeds, which includes the Utrerana breed, and it also disseminates the Andalusian poultry heritage in the municipalities of the province (including the transfer of specimens to the network of provincial rural lodgings) and the region in agreement with breeders’ associations [20,66]. For this purpose, conservation nuclei in purity are maintained for each variety of the Utrerana chicken breed, Black, Partridge and Black-barred, as well as specialised family lines being selected for obtaining capons and poulards in order to recover traditional gastronomy. It also organises technical conferences and participates with stands at local livestock fairs, as well as organising AVICOR, a poultry fair of autochthonous avian breeds. Activities for the formation of a germplasm bank for this breed were also planned [66]. León et al. [25] also mention the work of the Provincial Agricultural Centre of the Provincial Council of Cordoba in the development and extension of the characterisation and dissemination of Andalusian avian breeds basing it on ex situ conservation programs, highlighting the initiative to advance the recognition of the breed for its inclusion in the Official Catalogue of Livestock Breeds of Spain (in collaboration with breeders’ associations; see Section 15). On 1 June 2006, the Asociación Avícola Andaluza (Andalusian Poultry Association, in English) received the technical report of the proposal for the official recognition of the Utrerana breed, prepared by the veterinary technicians of the Provincial Agricultural Centre of the Provincial Council of Cordoba. Also noteworthy are the programs for the transfer of batches of birds to breeders in the province of Cordoba, as well as the teaching of bird castration courses.

Between the objectives of the Andalusian poultry genetic resources conservation program were the creation of a genealogical and productive database to implement a selection program aimed at improving laying performance of the Utrerana breed [20]. In fact, recently a broad functional characterisation of the Utrerana chicken breed has been undertaken using birds from the Provincial Agricultural Centre of the Provincial Council of Cordoba, which are described in detail in other sections (see Section 12, Section 13 and Section 14).

## 11. Recent Advances in Genetic Characterisation of the Utrerana Chicken Breed

When genetic research methodologies advanced, beginning in the late 2000s more genetic studies on this autochthonous breed were carried out, which are key to implement conservation strategies and to understand its relationships with other chicken breeds. The main results of these studies are summarised in this section.

The first of them [67,68] analysed the genetic diversity by using 24 microsatellite markers of 13 Spanish chicken breeds (including a synthetic one), a White Leghorn population and a recessive wheaten gene line that are kept at the El Encín Research Station within the framework of INIA’s conservation program (see Section 10.1). A total of 150 alleles were detected across all these breeds, 48% of them being missed in the White Leghorn. The mean expected heterozygosity was higher than the mean observed heterozygosity in the Partridge and Black-barred varieties of the Andaluza breed (H_e_ = 0.518 and H_o_ = 0.495, and H_e_ = 0.502 and H_o_ = 0.474, respectively), with higher values than the average of the breeds under study (H_e_ = 0.488 and H_o_ = 0.461). The number of alleles per locus was 3.500 in the Black-barred variety and 3.292 in the Partridge variety (average of all breeds: 3.405), showing an excess of heterozygotes (F_IS_ = 0.046 and 0.113, respectively). The number of loci that deviated from the Hardy–Weinberg equilibrium was two and four in the Black-barred and Partridge varieties, respectively (average of all breeds: 2.666), thus suggesting that they have been selected for a long time for morphological traits. Among all the breeds studied, Nei’s genetic distance was the smallest between the two varieties of the Utrerana breed (0.215) and largest between both varieties and the White Leghorn (0.352 for both varieties). In summary, a high degree of differentiation was observed among the breeds studied. The populations of the Partridge and Black-barred varieties from this program showed higher heterozygosity, presented a high number of specific alleles, and were more polymorphic than the White Leghorn population, suggesting their good potential for use in alternative production systems. Moreover, Dávila et al. [68] indicate that, despite the fact that Spanish chicken breeds have traditionally been selected for morphological characters, there is a clear separation between the White Leghorn and the Spanish breeds, indicating the importance of including these autochthonous populations in conservation programs.

Another study, carried out by researchers from various agricultural research and development institutions [69], characterised the genetic diversity and population structure of 13 Spanish autochthonous chicken breeds by using 30 microsatellites as genetic markers. Observed heterozygosity (H_o_) ranged between 0.40 and 0.57, with the White Utrerana variety showing the highest value, while unbiased expected heterozygosity ranged between 0.44 and 0.61. A network based on Nei D_A_ genetic distances, computed by means of the NeighborNet method, revealed that southern Spain breeds grouped together. In fact, following Bayesian cluster analysis, if the existence of two populations is assumed (K = 2), the chicken breeds of southern Spain differentiated from the rest of populations, while with K = 11 (the optimum), all breeds grouped independently, excepting the Black-barred and White varieties of the Utrerana breed and, therefore, the Partridge variety of the Utrerana breed maintained its own cluster. Moreover, the third axis of a factorial correspondence analysis differentiated Partridge Utrerana variety, Mallorquina and Andaluza Azul breeds.

A third study was carried out at the University of Lleida (Spain) and consisted of a comparative analysis between 19 chicken breeds from the Mediterranean group, 15 of them from Spain and four from Italy, based on their morphological characteristics, to carry out a systematic classification of the breeds in related groups [16]. To this end, 17 qualitative morphological characteristics were evaluated by means of the principle of parsimony using the Fitch method, obtaining a phylogram (which is not a phylogenetic tree) and a strict consensus tree. It was found that the analysis of the morphological characteristics does not reveal relationships of origin or aptitude among breeds. In addition, the greatest morphological similarity between the Black-barred and White varieties, on the one hand, and between the Partridge and Black varieties, on the other hand, was demonstrated for the Utrerana breed. On the other hand, according to the strict consensus tree, the Utrerana breed shows greater morphological similarity with breeds such as Menorquina, Penedesenca Trigueña, Andaluza Azul, Alicantina, Murciana, Valenciana de Chulilla, Padovana, Black Castellana, Cara Blanca, and a little less with Italiana, Livorno and Sobrarbe. On the contrary, the Utrerana breed shows less morphological similarity with Mallorquina, Pairal, Flor d’Ametller, Mericanel della Brianza, Prat, Penedesenca (Aperdizada, Negra, and Barrada Dorada varieties), Empordanesa (Aperdizada, Blanca, Blanquirrubia, Roja, and Rubia varieties) breeds.

A study on the genetic diversity of the four plumage varieties of the Utrerana chicken [27,70] was carried out by the Department of Genetics of the University of Cordoba and the IFAPA Centre of Cordoba by using 30 microsatellites on samples gathered from several breeders affiliated to the Asociación Nacional de Criadores de Gallinas Utreranas (ANCGU). The breed presents a moderate–high intrabreed genetic diversity and low consanguinity in its populations. The average observed and expected heterozygosity were H_o_ = 0.498 and H_e_ = 0.588, respectively. The fact that the population deviates from the Hardy–Weinberg equilibrium can be due to the existence of the four varieties, suggesting the existence of racial subdivision. Regarding the genetic structure, a factorial correspondence analysis showed that White, Black and Black-barred varieties constitute a group and the Partridge variety differs from the rest on axis 1, while axes 2 and 3 separate White variety from the rest; Black and Black-barred varieties conform a single group with the lower genetic distance between them. The genetic structure found in the Utrerana chicken does not correspond to the colour of the plumage. Thus, the tree of individual distances shows that it is not possible to differentiate groups based on plumage colour (although the Partridge variety groups better). The Utrerana breed is not a homogeneous population, since the Partridge variety is separated from the others when it is assumed that the number of ancestral populations is K = 2 (the optimum). When K = 3, the Partridge variety remains homogeneous while a subdivision is observed in the other three varieties that does not correspond to the colour of the plumage. When K = 4, a subdivision of the Partridge variety is observed.

Using Spanish chicken breeds from the INIA’s conservation program, research has been also carried on the association between polymorphism in the melanocortin 1 receptor gene (*MCR1*) and locus *E* plumage colour phenotype [71], which affects the distribution of eumelanin and phaeomelanin in feathers, being found as E*E (extended black in the Black-barred variety) and as E*N (wild type) in the Partridge variety of the Andaluza breed. Single-nucleotide polymorphism with allelic frequency ≥ 0.5 for the alleles of the *E* locus showed that the Black-barred variety had the G274A polymorphism, as carrying E*E alleles. The frequency of haplotypes of the *MCR1* gene in the different alleles of the *E* locus was higher for the H0 haplotype in the Partridge variety and for H4 haplotype in the Black-barred variety. The study confirmed that *E* locus is equivalent to *MCR1*.

## 12. Recent Characterization of the Productive Performance of the Utrerana Chicken

Research on the aspects most directly related to the productive potential of the Utrerana breed, both for eggs and meat, had previously received little attention from the scientific community possibly because, as already indicated, shortly after its consolidation as a breed it was relegated of intensive commercial poultry farming with the dispersion of select strains of chickens. To a large extent, this knowledge gap has recently been filled by a research project on a conservation strategy of the Utrerana chicken and valorisation of its products (“Estrategia de conservación de la Gallina Utrerana: valorización de sus productos”) funded by FEDER [72], developed by a large team of researchers from the IFAPA Centre of Cordoba, the Provincial Agricultural Centre of the Provincial Council of Cordoba and the Department of Genetics of the University of Cordoba. The main results of this project consisted of a doctoral thesis (“Functional characterization of varieties of Utrerana Avian Breed” [17]) and a wide production of scientific papers, mainly focussing on characterising biometry, reproductive performance, growth curves (see Section 13), egg laying curve and performance, egg quality and sensory evaluation (see Section 14) and other aspects of the Utrerana chicken breed. Some of these studies are summarised in this section.

A biometric characterisation of the Utrerana and Sureña breeds [73], carried out based on phaneroptic and other traits measured in birds reared under backyard regime by breeders from Andalusia, has confirmed the sexual dimorphism (due to sexual selection for larger roosters), the rustic character adapted to free-range rearing and the egg-laying potential of the Utrerana breed. In addition, it has confirmed that this breed is morphologically different from the Sureña chicken breed. This research showed that nail colour is the best discriminant trait to discriminate hens, permitting to discriminate among Black, Black-barred and Partridge Utrerana varieties, as well as Black Sureña; ocular ratio also helped in discriminating between hens, across the varieties of Sureña and Utrerana. However, roosters only differed depending on ocular ratio. Based on morphological traits, three clusters are defined in Utrerana breed: Black, Partridge and White and Black-barred varieties. The fact that White and Black-barred Utrerana varieties grouped closely suggests lingering effects of hybridisation between broth varieties, probably because breeders crossed them to decrease consanguinity in the scarce nuclei of the White variety.

A basic characterisation of the reproductive performance has revealed the good reproductive potential of the Utrerana chicken breed, which can be summarised as a fertility of 90.7% (lower in the Partridge variety), an embryonic mortality of 14.2% (lower in the White variety), perinatal mortality of 4.8% (lower in the Black variety) and an average chick weight at hatch of 47.2 g, which progressively decreases in the Partridge, White, Black and Black-barred varieties [29]. Another study has investigated the sexing of day-old chicks of the four varieties of the Utrerana breed [74], identifying four efficient early sexing methods, suitable regardless of variety: (i) English method, in which the chick is suspended by the beak or by the skin of the neck, and the posture it adopts is observed, so that females kick while males maintain a relaxed posture; (ii) general colouring of down feathers, which differs among varieties, but on the sides of the chick it is heterogeneous in females and homogeneous in males; (iii) wing fan, with wing feathers forming a homogeneous fan-shaped edge in females, while these feathers have heterogeneous lengths in males; and (iv) coping behaviour, in which the hands are clapped at 20 cm from the chick, placed in isolation, and males remain immobile while females show flight behaviour.

The laying curve, studied for a year, is characterized by the fact that peak hen-day production is reached at different times depending on the variety, ranging from 7 or 9 weeks after the start of laying in the Partridge and Black varieties, respectively, to 13 or 14 weeks in the White and Black-barred varieties, respectively. The hen-day percent egg production at the laying peak ranges between 50% for the White variety and 66% for the Partridge variety [18]. This agrees with a study carried out at the University of Seville reporting 71 hen-day percent egg production at laying peak in caged Utrerana hens of the Partridge variety [75]. It has also been shown that the persistence of egg laying after the peak differs among plumage varieties, with the Partridge variety experiencing a sharper seasonality. Moreover, the number of eggs laid per hen per year is 121 eggs in the Black-barred variety, 109 eggs in the White variety, 107 eggs in the Black variety and 94 eggs in the Partridge variety [18]. These results do not confirm higher egg yield values reported by informative publications on the breed [5,6]. Overall, the laying performance of the Utrerana breed is low, although it maintains acceptable laying rates during the hottest months of the year, thus showing potential towards some tolerance to heat stress [18]. It is interesting to note how, after abandoning productive farming and selection of the Utrerana breed after the irruption of improved white and brown layers that have dominated intensive poultry farming in Spain since the second half of the 20th century, the production of about 180 eggs per hen per year that reached the breed in its heyday fell back to the levels prior to its selection, of about 111 eggs per year [8]. It would be interesting to select and improve the breed for egg productivity to valorise it in alternative production systems, such as organic farming.

Within the framework of the aforementioned project, a crossbreeding trial of the Utrerana breed with heavy breeds (Brahma and Plymouth Rock) was also carried out in order to evaluate the possibilities of using males for caponisation, as a way of giving them a utility. The quality of this product and its acceptance by hospitality and restaurant professionals were evaluated [72].

## 13. Growth Curves

The Utrerana chicken can be considered, mainly, as an egg-laying-oriented breed. However, as with most autochthonous breeds, it is also bred for meat, particularly the male birds for self-consumption [31]. Hence, there is an interest in knowing its growth potential, which has led to investigating its growth curves. Three studies stand out in this regard, carried out within the framework of the conservation program of the Utrerana hen of the Provincial Agricultural Center of the Provincial Council of Cordoba.

In the first of them, Cabello et al. [76] fitted growth curves for the Black, Black-barred and Partridge varieties up to 15 weeks of age by using the Gompertz model. It was found that at that age the Black-barred variety reaches the highest mature weight (2870 g), followed by the Black variety (2504 g), with the Partridge variety achieving the lowest weight (2181 g) of the three varieties. The Black-barred variety shows the highest growth rate up to the inflection point of the growth curve, while the Partridge variety shows a lower growth rate from the inflection point. The Utrerana breed reaches the inflection point of the live weight curve at around two months of age, specifically at 62, 59 and 61 days in the Black-barred, Black and Partridge varieties, respectively. At these inflection points, the birds reach live weights of 1056, 922 and 803 g in the Black-barred, Black and Partridge varieties, respectively.

In a second study, González Ariza et al. [77] fitted growth curves separately for each sex up to 20 months of age. They do not indicate the variety or varieties of Utrerana chickens used in their trial. The Gompertz model showed the best adjustment to the growth of males and the Von Bertalanffy model for females. A clear sexual dimorphism of the breed was evidenced. The inflection point of the growth curve is reached earlier by females (58 days of age) than by males (73 days), weighing 529 and 846 g at that time, respectively. On the contrary, males show a greater precocity than females in reaching the weight at maturity; thus, the adult weight of males is 2300 g and that of females is 1786 g.

In a third study, González Ariza et al. [78] analysed the growth curves of each of the four plumage varieties of the Utrerana breed up to one year of age, reared under free-range conditions. For both sexes, the Logistic model was better adjusted to the growth of the White variety, while for the other three varieties the adjustment was better with the Von Bertalanffy model. The Black variety was the heaviest (maturity weight of 2606 and 2033 g for roosters and hens, respectively) while the White variety showed the lower maturity weight (2443 and 1874 g for roosters and hens, respectively). The maturity weights of the Black-barred variety are 2596 and 2024 g for roosters and hens, respectively, and in the Partridge variety these are 2484 g in roosters and 1938 in hens. Males show a higher body weight than females in all growing stages, although more evidently from 45 days of age. Utrerana can be defined as a light breed due to the early age at which it reaches the inflection point of the growth curve, the high precociousness and the low weight at maturity. Both sexes of the White variety show lower maturity weights, while for the other three varieties, both sexes display similar values. Maturity was reached later in the Black-barred variety, while an earlier growth was reported for the White variety.

The main conclusions of research on the growth curves of the Utrerana chicken are that, in accordance with its live weight and growth pattern, it can be considered a light [76,77,78], slow-growing breed [78], with an unimproved genotype oriented more to egg production, with clear sexual dimorphism [77] and that shows inter-varieties weight differences [78].

Given the current low live weight of the Utrerana breed, a program to improve growth would be desirable, which would entail an increase in adult weight, more time until the inflection point of the growth curve and maintenance of the growth slowdown [76]. In this regard, males of this breed could be profitable from a meat production point of view [78]. By developing a proper selection program, the breed would have the potential for meat production in alternative systems, such as organic farming, for which slow-growing genotypes are suitable, as they allow reaching slaughter weights appropriate to market demands at the minimum legal slaughter age, set at 81 days of age in the European Union (EU) [79].

Further research is needed to estimate the genetic parameters of the growth curve of this breed and make a genetic selection of individuals based on growth characteristics [78].

## 14. Characterization of Quality and Acceptance of the Utrerana Hen Eggs

Despite the fact that the Utrerana is considered a predominantly egg-oriented breed, research on aspects related to the quality of its eggs has not been undertaken until recently. The main studies in this area are summarized below.

The average values of the main external quality characteristics of the Utrerana hen’s egg, taking into account the four varieties, vary in the following ranges [17]: egg weight, 59.1–63.5 g; shape index, 71.3–75.6%; and eggshell thickness, 0.37–0.40 mm. The average values of the main internal quality characteristics are [17]: albumen, 52.4–56.6%; yolk, 28.4–32.3%; eggshell, 12.5–13.8%; and Haugh units, 82.2–86.9.

With regard to quality attributes, differences have been found between the Utrerana hen’s egg and the White Leghorn egg in most of the parameters, except for yolk diameter, albumen height, yolk pH and L* trichromatic coordinate of yolk colour. Hen age influences eggshell weight, egg width, albumen height and L* and a* trichromatic coordinates of yolk colour. The month of laying and the time of the laying period affect albumen height and yolk colour. Moreover, it was seen that egg external quality traits predict better internal quality ones than vice versa [80]. Regarding the relationship between internal and external quality traits and the commercial classification of egg grading by weight [81], it has been found that albumen, yolk, and eggshell weights are the most influential traits determining differences among egg quality categories. It has also been reported that, when egg quality is grouped depending of egg weight grading, the eggs of Partridge and Black-barred Utrerana varieties are confused with each other because they have larger yolks and slightly lower weights, and that there are similarities between the eggs of White Utrerana variety and White Leghorn due to the lingering effect of hybridisation between them [82].

A characterization of the external and internal quality of the Utrerana hen eggs of the Partridge variety during the first 12 weeks of the laying period, carried out at the University of Seville (Spain) [75], showed that the average egg weight was 62 g and it increased during the first three months of the laying period. Their shape index (71.2%) showed that the eggs are more elongated than those of commercial layer strains. The colour of the shell is white and the colour of the yolk is orange (10 points on average on the Roche scale). Eggs progressively lost freshness when stored at room temperature, with Haugh units declining between 82.7 the day after laying and 66.9 seven days after laying.

Regarding the chemical composition of the Utrerana hen’s egg, it has been reported that it contains more calcium in the shell (+6.4%) and in the albumen (+1.6%), more Pb (+5.7%), protein (+3.0%) and fat (+16.1%) in the yolk, more protein in the albumen (+2.9%), and more vitamin E (+15.9%), polyunsaturated and some monounsaturated fatty acids in the yolk than the egg of the White Leghorn. The cholesterol content of the egg yolk (1085 mg/kg) did not differ among the varieties of the Utrerana breed or the White Leghorn breed. In addition, the linoleic acid content in the yolk is higher in the Black Utrerana variety and White Leghorn than in the Black-barred and Partridge Utrerana varieties [83].

A sensory evaluation with hospitality and restaurant professionals and students has also been carried out by comparing eggs from the Utrerana breed and White Leghorn (including organic) eggs. Utrerana hen eggs were more appreciated than those of the White Leghorn and organic eggs in aspects such as yolk colour, odour, flavour, texture and global acceptance, and the visual appearance of the whole egg and the cracked egg on a plate was better valued. Moreover, the professional profile influences sensory attributes’ assessment and the consumption of eggs [84].

This set of studies have evidenced the excellent quality of the egg of the Utrerana breed, which can be used to value the breed among consumers. In fact, a study carried out to compare the quality of the eggs of the Utrerana breed varieties, four other native Spanish breeds (Andaluza Azul, Cara Blanca and White and Black varieties of the Tufona breed), the Araucana breed and the White Leghorn has shown that eggs can be differentiated between genotypes based on the trichromatic coordinates a* and b* of the eggshell, and that the Mediterranean breeds of hens differ from the White Leghorn based on egg quality parameters [85].

## 15. Official Recognition and Breeding Program of the Utrerana Chicken Breed

The first attempt to officially recognise of the Utrerana chicken breed was made in the Committee of Livestock Breeds of Spain held on 14 June 2005, but the proposal did not prosper due to lack of consensus among the representatives of various Autonomous Communities. After taking the initiative from Andalusia, with the participation of the Asociación Andaluza de Avicultura, and once a new technical report by the Agricultural Provincial Center of the Provincial Council of Cordoba (see Section 10.2) had been prepared, the recognition of the Utrerana breed was approved at the meeting of the Committee of Livestock Breeds of Spain held on 21 June 2006 [25]. Therefore, the Utrerana chicken has been included in the Official Catalogue of Livestock Breeds of Spain, with the category of autochthonous breed, after the publication of Orden APA/53/2007 on 17 January 2007 [15]. Current version of the Official Catalogue was published by means of Real Decreto 527/2023 [2], which updated Real Decreto 45/2019 [86].

The Utrerana Breed Breeding Program was approved on 11 December 2019 by the General Directorate of Agricultural and Livestock Production of the Regional Government of Andalusia [33]. It describes the participants in this breeding program (in addition to breeders: the Department of Genetics of the University of Cordoba and the Agricultural Provincial Centre of the Provincial Council of Cordoba), the censuses, the breed standard (see Section 5) and bird qualification system, the Herd-book structure and instructions to register birds and farms, the Breeding Program, and the structure of the management committee of the Breeding Program.

According to the Breeding Program, the morphological qualification of birds is carried out by the method of scoring body regions, scoring each region from 1 to 10 points and weighting them with the following coefficients: head, 2; neck, 1.5; back and breast, 1.5; tail, thigh and legs, 1.5; plumage, 1.5; and general condition, 2. The assignment of less than 5 points to a region implies the disqualification of the bird. The final score, which is obtained by multiplying the rating of each region by its weighting coefficient, allows birds to be classified as: Poor (<70 points), Sufficient (70 to <75 points), Good (75 to <80 points), Very good (80 to <90 points) or Excellent (≥90 points).

All birds registered in the Herd-Book must be individually identified with a numbered ring or wing tag.

The Herd-Book is made up of a Supplementary Section and a Main Section. In the Supplementary Section are registered: (a) birds with completely or partially unknown genealogy and (b) males and females of which at least one of the parents is unknown but have a definitive identification and have achieved a morphological qualification of at least Sufficient (≥70 points), proving their potential as breeding birds. The Main Section consists of: (a) a Basic Class, which includes birds for the registration of which only genealogical requirements are necessary, that is, males and females descended from breeding birds registered in the Main Section or in the Supplementary Section; and (b) a category of Breeding Birds with Merits, which includes birds of both sexes from the Main Section that have some merit, such as having obtained at least 95 points in conformation shows or having passed the tests for their inbreeding coefficient and their genetic conservation index, as well as other genetic-functional tests established in the Breeding Program.

The Herd-Book also includes the farms registry and the genealogical record, which is based on the filiation declaration, made between 7 and 9 months of age of the birds.

The general objective of the Breeding Program is to function as a conservation program by maintaining the genetic diversity of the breed, and it has two specific objectives: (1) maintenance of the levels of genetic diversity of the breed (through two criteria: value of the individual inbreeding coefficient and value of the coancestry coefficient of scheduled matings); and (2) maintenance of the levels of genuineness of the breed (through the criterion of the individual genetic conservation index value—average founder effect).

On 14 December 2021, the Program for the Diffusion of the Genetic Improvement of the Utrerana Breed was approved by the General Directorate of Agricultural and Livestock Production of the Regional Government of Andalusia [87]. It includes the following measures: (i) zootechnical advice to farms; (ii) training for farmers; (iii) promoting scientific, technical and informative publications on the breed, as well implementing programs to disseminate the breed and its products and benefits; (iv) for the genetic connection of farms, it is intended to put into operation a program for the distribution of fertilized eggs while promoting the transfer of breeding birds among the farmers that contributes to optimise the operation of the Breeding Program. Based on the genetic diversity studies of the breed, breeders will be recommended which will be the most suitable pairings. A germplasm bank will be set up; (v) the breeders’ Association (ANCGU) will participate in select livestock competitions; (vi) ANCGU will be in charge of coordinating actions related to the sale of breeding stock and fertilized eggs; and (vii) a social awareness plan will be established aiming to reintroduce the breed into the productive sector, through the implementation of dissemination actions in agritourism companies, farm schools and environmental education centres, as well as in the field of owners of small-capacity and/or self-consumption farms.

## 16. The Breeders’ Association: “Asociación Nacional de Criadores de Gallinas Utreranas” (ANCGU)

As indicated in Section 6 and Section 10.2, the role of amateur breeders has been key in the conservation of the Utrerana chicken breed once it lost its use in commercial poultry farming. In the first stage, they were organized in the Asociación Andaluza de Avicultura, which supported the first steps for the recovery and official recognition of the breed [5,25].

The current association of breeders of this avian breed, Asociación Nacional de Criadores de Gallinas Utreranas (ANCGU), was founded on 4 February 2009 by a group of amateur poultry breeders from Utrera (Seville province) led by Mr. Juan Manuel Sánchez Ocaña, being officially approved on 28 October 2009 [24]. It currently has 55 members distributed throughout the Spanish provinces of Seville, Cordoba, Huelva, Cadiz, Málaga, Guadalajara, Toledo and Tenerife. The purposes of the association are:

- The breeding, selection and promotion of the Utrerana chicken in all its varieties;
- Breed conservation.

And to achieve these objectives, it develops the following activities:

- The management of the Herd-Book;
- The genetic improvement of the breed;
- The dissemination of the breed.

On 4 December 2013, the General Directorate of Agricultural and Livestock Production of the Regional Government of Andalusia officially recognised the Asociación Nacional de Criadores de Gallinas Utreranas as the organisation of purebred Utrerana chicken breeders in charge of the keeping of the Herd-Book of the breed [26,33].

The ANCGU organised several Utrerana Chicken Fair events (see Section 17) [88,89,90,91,92,93,94,95], and efforts are currently being made to renew the celebration of this Fair. Moreover, ANCGU also participates in other poultry fairs (e.g., “Feria Avícola Sierra de Cádiz-Villa de Zahara”, “FEGASUR”, “Una pará en Gines”, etc.). In addition, the Association has organised several conferences and seminars on the breed.

Activities that the ANCGU is undertaking are (i) collaborating with the Savory Institute for the holistic management of poultry farms; (ii) guiding farms to improve the environment, ecological production and the sustainability of their livestock products; (iii) promoting self-consumption farms; (iv) valorising the rural and artisanal market for Utrerana chickens and their products so that the income of poultry farmers can be sufficient for the maintenance of this type of farm; and (v) promoting a return to the classic mating carried out by the creators of the breed.

On the occasion of the centenary of the foundation of the breed, the ANCGU plans to hold a series of events in 2026 for the promotion and valorisation of the Utrerana hen, aimed also at an international projection and to insert the breed and its farms in the tourism circuit of Utrera.

## 17. The Utrerana Chicken Fair as a Promotion Forum for the Utrerana Chicken Breed

The organisation and celebration of the Utrerana Chicken Fair (“Feria de la Gallina Utrerana”, in Spanish) has contributed in a particularly relevant way to the dissemination of the breed, especially among the general public and amateur breeders. The Fair, co-organized by the Utrera City Council and the Asociación Nacional de Criadores de Gallinas Utreranas (ANCGU), and supported by companies in the sector, began in 2004 and was held annually for 15 years until 2018.

The Fair aimed at promoting the knowledge, exhibition and dissemination of the four varieties of this breed and included various activities, such as the exhibition and sale of live birds, conferences on the breed and its conservation and the celebration of the conformation shows of the breed. Table 4 shows the list of birds presented to the conformation shows held in the framework of the Fair between 2006 and 2014.

The number of birds participating in the conformation shows of this Fair increased rapidly, so that in the fifth edition it already exceeded 60 animals, and the presence of hens prevailed over roosters. By varieties, the Partridge predominated, followed by the Black and, finally, the Black-barred variety. In addition to the birds listed in Table 4, in the 2014 edition of the Fair, three roosters of the White variety also participated in the conformation show [95]. The scarce presence of the White variety in the conformation shows is due to its low population.

## 18. Evolution of the Census of Birds and Farms of the Utrerana Chicken Breed Registered in the Herd-Book of the Breed

Table 5 shows the evolution of the census of birds of Utrerana chicken breed registered in the Herd-Book during 2014–2022, according to the information of the Official Catalogue of Livestock Breeds of Spain [7].

As can be seen in Table 5, and according to the information from the Herd-Book, the evolutionary trend up to 2022 of the number of active farms, the total number of birds and the number of hens of the Utrerana breed was towards expansion [7]. Most of the farms and birds registered in the Herd-Book are located in Andalusia, with one farm in the Castile–La Mancha region. Moreover, out of the 1822 birds registered in the Herd-book in 2022, 8.5, 10.6, 32.4 and 35.0% were of the White, Black-barred, Black and Partridge varieties, respectively [33], which illustrates the relative abundance of each variety.

Table 6 shows the evolution in the provinces of Andalusia of total census of birds of Utrerana chicken breed registered in the Herd-Book during 2014–2022. It can be seen that during this period, the census has expanded from an almost exclusive location in the province of Seville to a broader distribution in almost all of Andalusia, but with 75% concentrated in the provinces of Seville and Cordoba, and with an absence of birds in the province of Almeria.

González-Ariza et al. [72] report that the census of birds registered in the Herd-Book increased considerably, especially from 2018, when the distribution program of birds among the breeders of ANCGU from the project “Estrategia de conservación de la Gallina Utrerana: valorización de sus productos” began (see Section 12), managing to break the almost exclusive encapsulation of the breed population in the province of Seville and expanding it to almost all Andalusia. This allowed the dissemination of the breed and the increase in the number of birds to avoid its extinction [72].

In summary, after its initial expansion following its creation and selection, and after going into decline in the second half of the 20th century when it was relegated from productive poultry farming with the dispersion of improved foreign breeds, the population of the Utrerana breed is currently in a phase of progressive recovery due to the praiseworthy efforts of amateur breeders, with most of the census located in Andalusia.

## 19. Logo “Autochthonous Breed”

The Asociación Nacional de Criadores de Gallinas Utreranas has been granted the “Autochthonous Breed” Logo (“Logotipo Raza Autóctona”, in Spanish) since 2021 to apply it to its products [97]. According to the bid specifications of this quality logo, the scope of application to the products obtained from the Utrerana chicken breed are all the operators of the value chain, from birth of the birds to the sale of the product to the consumer: it is applied to primary production (poultry farms), transformation (slaughterhouses, meat-cutting rooms and packaging centres) and marketing, including restaurants where products of the Utrerana breed are consumed. The products authorised for the use of the “100% Utrerana Autochthonous Breed” denomination are meat and eggs, as well as manufactured or processed products, in which one of the main ingredients is the meat or eggs of the breed. It may also be used in meals prepared with products of the breed.

The eggs obtained from the hens of the breed must come from farms registered in the Herd-Book. In the event that birds of other breeds exist on these farms, the flocks of the Utrerana breed must be raised completely separated from the other chickens. In this case, the collection and storage of the eggs will be carried out separately, avoiding mixing eggs, being identified and delivered in differentiated packaging. In the case of sale in cases, each egg carton will bear, in addition to the identification of the farm, an adhesive security label of the Association with specific numbering associated with the farm of origin. The sale of eggs may benefit from the regulation of the administrative regime and the information system of direct sale of primary products from farms to final consumers and retail establishments. When the current regulations on the marketing of eggs allow exceptions to the mandatory marking, at the point of sale the eggs must be completely separated from those of other breeds or strains, and in this case an ANCGU sign must appear indicating the data of the farm, the name and address of the owner, the Official Registry of Livestock Farms (“REGA” in Spanish) farm code and the breeder’s code in the Herd-Book.

Meat products admitted are: (i) *Utrerana breed rooster*: slaughtered male over 5 months of age and, in any case, provided that symptoms of the beginning of sexual activity are detected; (ii) *Utrerana breed hen*: female sacrificed for consumption with a minimum of 2 years of life, after completing at least one and a half annual laying periods; and (iii) *Utrerana breed capon*: male castrated at 2 months of age and sacrificed when aged over 5 months. For these three specific products, the following rules must be followed: It is sold with its head and legs. The carcass will be identified with a guarantee seal placed on the leg, with a specific numbering and identification of the product type, in red colour with black letters for *roosters*, in green colour with white letters for *hens* and in blue colour with white letters for *capons*. The reference number will be associated with the farm of origin.

The manufactured or transformed products must have as the main or one of the ingredients the meat or eggs of the breed. The following products are included: (a) processed meat products such as sausages, pepperoni, chorizos, soups, broths, etc.; (b) egg products; (c) prepared food resulting from the raw or cooked or precooked preparation of a meat product or eggs of the breed. All these products must bear an adhesive safety label from the Association with specific numbering, associated with the farm of origin.

The breeding conditions for live birds required by this quality logo are: (i) they must be registered in the Herd-Book and comply with the provisions of the Breeding Program; (ii) birds must be on farms registered in the Official Registry of Livestock Farms (REGA); (iii) farms must have a sanitary program to control infectious and parasitic processes, which will include a specific self-control sanitary program for the prevention and control of salmonellosis, as well as a code of good hygiene practices; (iv) the breeding for the commercialization of meat or eggs must comply with the Spanish and Community regulations for poultry farm management; and (v) the owners of organic farms and farms that have been authorised to run a traditional free-range or free-range farming system may avail themselves of the exception whereby certain conditions of application of the provisions of the EU are made more flexible in hygiene matter for the production and marketing of food products.

For the application of this logo, a traceability system is mandatory, which guarantees the existence of a complete record of the entire productive life of the bird, in which the bird or the product must be identified, through its labelling, at any point in the production and marketing chain. In addition, the labelling of products from the Utrerana breed must comply with the regulations for the marketing of eggs or poultry meat in force in the EU. All products and their presentations from birds must be accompanied by mandatory information in the form of a label. The “100% Utrerana Autochthonous Breed” logo will accompany the labelling by a reference number in all stages of production process.

The production area of the Utrerana breed, whose products are suitable to be covered by this logo, is made up of all the farms registered in the Herd-book and that belong to the Spanish territory, including the Autonomous Cities of Ceuta and Melilla [97].

This seal of quality favours the differentiation in the market of the products of the Utrerana breed. In this sense, the ANCGU is working to open short market channels to offer the consumer these products of proven quality and environmental sustainability, and that make profitability recovery possible for small breeders of Utrerana chicken.

## 20. Challenges and Threats Faced by the Utrerana Chicken Breed

Like other native breeds that are in a similar situation, the Utrerana chicken breed faces a series of threats and challenges that limit its expansion, of which the following stand out:

- The small number of breeders willing to undertake productive breeding aimed at marketing eggs or meat, due to the bureaucratic obstacles it entails, constitutes a threat, and it would be advisable to address the challenge of exploring this route as a way of creating complementary farms that would allow the expansion of the breed and provide additional income to breeders;
- Another threat is the limited use of this breed in both conventional and organic farms due to its low egg and meat production performance compared to industrial breeds and strains, which makes it not economically profitable. Undertaking genetic improvement programs for the breed to increase its productivity is a pending challenge;
- A threat derived from the small number of birds kept by breeders is the difficulty of finding pullets of the Utrerana breed in sufficient numbers for free-range and organic poultry farming, something necessary to constitute homogeneous flocks with a sufficient farm size, which makes that producers choose to acquire other commercial breeds of which there is enough supply and availability in multiplication farms. The challenge of any multiplication farm specialised in alternative poultry farming undertaking a breed multiplication program, associated with a genetic improvement program suggested in the previous point, should be addressed;
- The growing legal and bureaucratic requirements that limit the uncontrolled possession and sale of animals constitute a threat to some amateur breeders who do not have their farms registered, which may cause some of them to abandon the breed raising. The challenge should be undertaken by the administration and the ANCGU to promote these amateur breeders to register their farms.

## 21. Perspectives of the Utrerana Chicken Breed and Future Actions

This bibliographic review has revealed the existence of several knowledge gaps related to the Utrerana chicken breed, which should be investigated in the future:

- Until now, a characterisation of the breeders of the Utrerana chicken breed had not been carried out in relation to describing the size of the farms and the varieties of birds they breed and the characteristics of their facilities and management, as well as other aspects related to commercialisation of the products. This gap is currently being filled by co-author Antonio Plata-Casado’s doctoral thesis at the University of Seville (Spain), which is in preparation;
- One aspect that has not received attention in the field of research is the carcass and meat quality, possibly because the Utrerana chicken has been considered a mainly egg-oriented breed. In fact, informative literature has been published reporting that the meat of the Utrerana chicken has an excellent flavour, being of good texture and not fibrous, with a white colour in the Black-barred variety and red in the Black and Partridge varieties [6]. Breeders also refer to the existence of differences in meat flavour between varieties of the breed (Mr. Juan Manuel Sánchez Ocaña, pers. comm.);
- In order to promote strategies that allow increasing the dissemination of the Utrerana chicken breed among more potential breeders and the consumption of its products among the public, it would be interesting to undertake research on the knowledge that the local and Andalusian population has about this breed and its products, to be carried out using survey methodology;
- Organising laying contests as a way of involving breeders in the improvement of the Utrerana chicken breed oriented towards egg production;
- Investigating the willingness of breeders to undertake production and marketing of the Utrerana chicken products, mainly eggs and meat, taking into account that this entails greater bureaucratic obstacles and zootechnical and sanitary controls.

In this sense, the ANCGU, the Utrera City Council and other agents are involved in formulating a strategy for the valorisation of the Utrerana chicken that aims to promote several of the aforementioned actions [98].

## 22. Conclusions

The Utrerana, a Mediterranean-type chicken breed, is autochthonous of southern Spain and presents particularities that make it an interesting animal genetic resource to preserve. It was created in 1926 by a poultry farmer from Utrera (Seville province) starting from local hens with a large body size that laid large eggs, selecting the breed first to achieve good egg-laying yields and later for morphology to obtain four plumage varieties: White, Black, Black-barred and Partridge. By the end of the second quarter of the 20th century, it was widely distributed and became competitive in the commercial egg laying industry in Spain. Subsequently, a population decline began in the second half of the 20th century that led the breed to be currently in danger of extinction, having been displaced from productive poultry farming by the proliferation of improved foreign breeds and hybrids that dominate current intensive poultry farming. The Utrerana chicken breed has been preserved to this day thanks to amateur breeders who have kept it in backyard systems for the self-consumption of its products and for its aesthetic values, its improvement for productivity being stopped. The work of these breeders and the implementation of conservation programs have led to the recent official recognition of the Utrerana chicken breed and to slowdown the decline in their censuses. Currently it is distributed mainly throughout the Andalusia region, with the White variety being the scarcest and the Partridge the most abundant. Its genetic and zootechnical characterization has shown that it is a light breed with sexual dimorphism and high genetic diversity, showing a functional orientation for egg laying and presenting traits of having been recently selected for conformation instead of productivity. The breed is characterised by a moderate laying performance with a tendency for seasonality, producing large eggs of good external and internal quality. It shows morpho-functional differences among varieties. Its growth is slow and has a rustic character, which makes it a suitable breed for alternative, free-range and organic production systems. This work has detected the gaps in knowledge about the breed to be covered, such as research on carcass and meat quality, as well as the need to valorise the breed among breeders and the population as a way of promoting its conservation.

## Figures and Tables

**Table 1:** Breed standards of the Utrerana chicken breed. Part 1: characteristics common to the four varieties. Adapted from del Castillo [8], Liaño [9], and Asociación Nacional de Criadores de Gallinas Utreranas [33].

Live weight:	Rooster:	3.000 kg
	Hen:	2.300 kg
Rooster shape:	Head:	Moderately long and wide. Rather thick.
	Beak:	Medium and strong.
	Eyes:	Large, bright.
	Comb:	A little larger than medium size. Not big. Deeply serrated and with five or six points. The rear point should slightly follow the line of the neck.
	Wattles:	Long, wide, slender and rounded at the bottom.
	Ear lobes:	Medium size.
	Neck:	Rather long. Gracefully curved.
	Wings:	Long and clinging to the body.
	Back:	Long, wide and straight. Quite sloped from shoulders to tail.
	Body:	Long and deep. Wide in the back and somewhat narrower in the saddle.
	Down:	Short and tight.
	Breast:	Wide, deep and well rounded. Carried upright and forward.
	Tail:	Raised and not very long. Sickle feathers well curved.
	Thighs:	Tall and robust.
	Shanks:	Rather long and thick. Free of feathers. Hocks are fully visible below the body line.
	Toes:	Four in number, straight and thin.
	Posture:	Graceful and elegant, denoting vigour and aggressiveness.
Hen shape:	Same morphological characteristics as the rooster, except for differences due to sex.	
	The comb falls to one side of the head, covering one eye.	

**Table 2:** Breed standards of the Utrerana chicken breed. Part 2: specific characteristics of each variety. Adapted from del Castillo [8], Liaño [9] and Asociación Nacional de Criadores de Gallinas Utreranas [33].

White	Black-Barred	Black	Partridge
*Colour:*			
Ear lobes and entire plumage: white.	Plumage: barred, made up of greyish white and dark grey stripes, which alternate in each feather in the form of transversal bars.	Plumage: black with greenish metallic reflections.	Comb, face and wattles, bright red.
Beak, shank and toes: rosy white.	Ear lobes: pure white.	Ear lobes: pure white.	Ear lobes: pure white.
Comb, face and wattles, bright red.	Comb, face and wattles, bright red.	Comb, face and wattles, bright red.	Eyes: reddish brown.
Eyes: reddish brown.	Eyes: reddish brown.	Eyes: reddish brown.	Beak: horny.
Hen eggs: white. Shades of cream colour are permitted.	Legs, toes and beak: rosy white.	Beak: black or horny.	Legs and toes: slate grey.
	Hen eggs: white. Shades of cream colour are permitted.	Legs and toes: black or slate black.	Hen eggs: white. Shades of cream colour are permitted.
		Hen eggs: white. Shades of cream colour are permitted.	Plumage is described in Table 3.
*Disqualifications:*			
(a) 300 g or more below the indicated weight.	(a) 300 g or more below the indicated weight.	(a) 300 g or more below the indicated weight.	(a) 300 g or more below the indicated weight.
(b) Red in the ear lobes in more than a third of its surface.	(b) Red in the ear lobes in more than a third of its surface.	(b) Red in the ear lobes in more than a third of its surface.	(b) Red in the ear lobes in more than a third of its surface.
(c) Spots of any colour on the plumage, tolerating the yellowish tone produced by the sun on the hackle and saddle feathers of adult roosters.	(c) Red or gold patches on plumage and all-white or all-black feathers.	(c) Spots of any colour on the plumage, white being tolerated on the wing feathers of the pullets before the first moult.	(c) White on the main tail feathers, sickles and secondaries.
(d) Any colour on beak, shanks and toes other than white or rosy white.	Note: The presence of grey spots on the legs, toes and beak does not constitute disqualification.	(d) Any colour on beak, shanks and toes other than black or slate black.	(d) Any colour on beak, shanks and toes other than slate grey.

**Table 3:** Breed standards of the Utrerana chicken breed. Part 3: Characteristics of the plumage of the Partridge variety. Adapted from del Castillo [8], Liaño [9], and Asociación Nacional de Criadores de Gallinas Utreranas [33].

Rooster		Hen	
Anatomic Region	Colour	Anatomic Region	Colour
Head:	Dark red.	Head:	Golden yellow with dark end.
Hackles:	Yellowish red with black spear tip in the middle of each feather.	Neck:	Golden yellow with black spear tip in the middle of each feather; neck front, salmon.
Neck front:	Glossy black.	Back:	The visible part of the feathers, light brown, dotted with the same darker colour, without grey or red tones.
Wings:	Front, black; bow, bright red; covers, greenish black; primaries dull black; secondaries, black with matt mahogany red on lower clothing.	Wings:	Bow and covers, equal to the back; primaries, slate brown; secondaries, brown, with dotted outer clothing of the same colour, lighter.
Back and saddle:	Bright red with a black spear tip in the middle of each feather.	Tail:	Matt black; the two highest main tail feathers may be dotted with brown; the covers are equal at the back.
Tail:	Black with greenish metallic reflections, brighter on the sickles and covers.	Breast:	Salmon colour, which lightens as it descends to the lower part of the body.
Breast, body and thighs:	Greenish black.	Body:	Light brown dotted with the same colour, darker.
Subcolour and down:	Slate grey. The down visible in the tail insertion, very light grey, almost white.	Thighs:	Slate grey heavily tinged with brown.
		Down:	Same as thighs.
		Subcolour:	Slate grey.

**Table 4:** List of birds of the Utrerana chicken breed presented to the conformation shows of the Utrerana Chicken Fair between 2006 and 2014 (*) [88,89,90,91,92,93,94,95].

Edition	Year	Black Variety		Partridge Variety		Black-Barred Variety		Total
		Roosters	Hens	Roosters	Hens	Roosters	Hens	
3	2006	4	4	7	4	1	-	20
4	2007	5	9	4	8	4	8	38
5	2008	5	23	9	17	2	8	64
6	2009	7	19	5	30	3	12	76
7	2010	10	17	11	10	6	16	70
8	2011	11	14	8	12	10	5	60
9	2012	4	13	6	14	7	5	49
11	2014	7	6	14	-	-	18	45

* The information for the 2013 Fair is missing.

**Table 5:** Evolution of the census of birds of Utrerana chicken breed registered in the Herd-Book during 2014–2022 (*). Adapted from Ministerio de Agricultura, Pesca y Alimentación [7].

**Year**	**Region**	**Foundational Registry**		**Births** **Registry**		**Definitive** **Registry**		**Total Breeding Birds**		**Total Birds**		**Total**	**Farms in Herd-Book**	**Average Farm Size**
		**H**	**R**	**H**	**R**	**H**	**R**	**H**	**R**	**H**	**R**			
2014	AND	56	15					56	15	56	15	71	7	10.1
2015	AND	133	25					133	25	133	25	158	12	
	MAD	7	4					7	4	7	4	11	1	
	Total	140	29					140	29	140	29	169	13	13.0
2016	AND	346	68	8	1			346	68	345	69	423	12	
	C-M	7	4					7	4	7	4	11	1	
	Total	353	72	8	1			353	72	361	73	434	13	33.4
2017	AND	609	129			7	1	616	130	616	130	746	19	
	C-M	7	4					7	4	7	4	11	1	
	Total	616	133			7	1	623	134	623	134	757	20	37.9
2018	AND	856	193	198	47	3	1	859	194	1057	241	1298	41	
	C-M	7	4					7	4	7	4	11	1	
	Total	863	197	198	47	3	1	866	198	1064	2045	1309	42	31.2
**Year**	**Region**	**Supplementary Section**		**Basic Class**		**Main Section Breeding Birds with Merits**		**Total Breeding Birds**		**Total Birds**		**Total**	**Farms in Herd-Book**	**Average Farm Size**
		**H**	**R**	**H**	**R**	**H**	**R**	**H**	**R**	**H**	**R**			
2019	AND	43	7	273	100	907	207	950	214	1223	314	1537	48	
	C-M					7	4	7	4	7	4	11	1	
	Total	43	7	273	100	914	211	957	218	1230	318	1548	49	31.6
2020	AND	203	36	192	59	847	188	847	188	1242	283	1525	49	
	C-M					7	4	7	4	7	4	11	1	
	Total	203	36	192	59	854	192	854	192	1249	287	1536	50	30.7
2021	AND	305	79	175	52	864	206	1334	337	1334	337	1681	50	
	C-M					7	4	7	4	7	4	11	1	
	Total	305	79	175	52	871	210	1351	341	1351	341	1692	51	33.2
2022	AND	391	125	175	52	864	206	1430	381	1430	381	1811	55	
	C-M			7	4			7	4	7	4	11	1	
	Total	391	123	182	56	864	206	1437	385	1437	385	1822	56	32.5

* As of 2019, the way of registering birds has been modified to adapt to requirements of Regulation (EU) 2016/1012 [96]. AND: Andalusia. C-M: Castile–La Mancha. MAD: Madrid. H: hens. R: roosters.

**Table 6:** Evolution in the provinces of Andalusia of total census of birds of Utrerana chicken breed registered in the Herd-Book during 2014–2022. Adapted from Ministerio de Agricultura, Pesca y Alimentación [7].

Province	Year								
	2014	2015	2016	2017	2018	2019	2020	2021	2022
Almeria	-	-	-	-	-	-	-	-	-
Cadiz	5	10	185	90	128	129	85	110	127
Cordoba	-	-	-	146	429	380	380	463	463
Granada	-	-	-	-	16	42	42	44	44
Huelva	-	-	-	-	36	28	28	43	43
Jaen	-	-	-	-	96	106	106	168	168
Málaga	-	-	-	30	30	30	30	48	48
Seville	66	148	238	480	563	822	854	805	918
*Total*	71	158	423	746	1298	1537	1525	1681	1811

## Data Availability

Data are contained within the article.
